# Assessing Patient Health Dynamics by Comparative CT Analysis: An Automatic Approach to Organ and Body Feature Evaluation

**DOI:** 10.3390/diagnostics14232760

**Published:** 2024-12-08

**Authors:** Dominik Müller, Jakob Christoph Voran, Mário Macedo, Dennis Hartmann, Charlotte Lind, Derk Frank, Björn Schreiweis, Frank Kramer, Hannes Ulrich

**Affiliations:** 1 IT-Infrastructure for Translational Medical Research, University of Augsburg, 86159 Augsburg, Germany; 2 Institute for Digital Medicine, University Hospital Augsburg, 86156 Augsburg, Germany; 3 Institute for Medical Informatics and Statistics, Kiel University and University Hospital Schleswig-Holstein, 24105 Kiel, Germany; 4 Department of Cardiology, Kiel University and University Hospital Schleswig-Holstein, 24105 Kiel, Germany; 5 German Centre for Cardiovascular Research, Partner Site Hamburg/Kiel/Lübeck, 24103 Kiel, Germany; 6 Medical Data Integration Center, University Hospital Schleswig-Holstein, 24105 Kiel, Germany

**Keywords:** radiomics, diagnostic imaging, computer tomography, health dynamics

## Abstract

**Background/Objectives:** The integration of machine learning into the domain of radiomics has revolutionized the approach to personalized medicine, particularly in oncology. Our research presents RadTA (RADiomics Trend Analysis), a novel framework developed to facilitate the automatic analysis of quantitative imaging biomarkers (QIBs) from time-series CT volumes. **Methods:** RadTA is designed to bridge a technical gap for medical experts and enable sophisticated radiomic analyses without deep learning expertise. The core of RadTA includes an automated command line interface, streamlined image segmentation, comprehensive feature extraction, and robust evaluation mechanisms. RadTA utilizes advanced segmentation models, specifically TotalSegmentator and Body Composition Analysis (BCA), to accurately delineate anatomical structures from CT scans. These models enable the extraction of a wide variety of radiomic features, which are subsequently processed and compared to assess health dynamics across timely corresponding CT series. **Results:** The effectiveness of RadTA was tested using the HNSCC-3DCT-RT dataset, which includes CT scans from oncological patients undergoing radiation therapy. The results demonstrate significant changes in tissue composition and provide insights into the physical effects of the treatment. **Conclusions:** RadTA demonstrates a step of clinical adoption in the field of radiomics, offering a user-friendly, robust, and effective tool for the analysis of patient health dynamics. It can potentially also be used for other medical specialties.

## 1. Introduction

Radiomics has experienced rapid growth in the last decade and empowered personalized medicine [1,2], especially in the field of oncology [3], by bridging the gap between radiology and cancer treatment [2,4,5,6,7,8]. Currently, it is extending its influence to various other medical fields [9]. The image-based computation of biomarkers has the potential to uncover biological patterns and characteristics that were previously undiscovered [10].

Key elements of radiomics are quantitative imaging biomarkers (QIBs), which serve as objective measures extracted from an in vivo image to accurately reflect normal biological or pathogenic processes, or even the body’s response to a particular treatment [11]. In contrast to qualitative imaging biomarkers, QIBs are particularly suitable for patients’ follow-up treatment. Due to strong advancements in automatic body segmentation and composition analysis through the recent development of deep learning-based tools, the effective and reliable computation of QIBs is now possible. 

However, a majority of existing tools are developed for computer scientists and deep learning specialists. This is a barrier for medical experts who are supposed to use and apply them clinically [12]. The three main issues are (1) selecting and setting up the right model, (2) interpreting the resulting data, and (3) trusting the information generated.

Our objective is to create and share a framework for the automatic calculation and comparison of time-series-based quantitative imaging biomarkers, with low technical barriers, that are visually comprehensible for medical experts.

## 2. Materials and Methods

**Figure 1:**
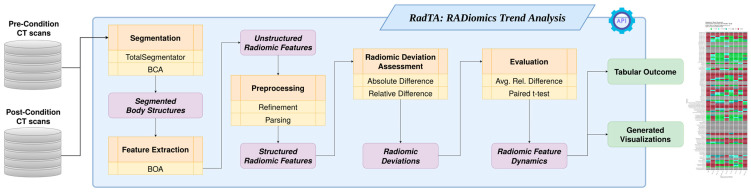
A workflow diagram of the RadTA framework.

In this section, we introduce our novel framework RadTA: RADiomics Trend Analysis [13]. RadTA is designed for clinical data scientists to conduct time-series-based radiomics trend analyzes on pre- and post-condition CT volumes. Our framework comprises six core components: a command line interface for usability, automated structure segmentation, radiomic feature extraction, the preprocessing of these features, the computation of radiomic feature deviation, and the evaluation of radiomic dynamics between pre- and post-condition states. Figure 1 illustrates the workflow diagram of RadTA, providing a visual representation of the framework’s components and their interactions. The following subchapters will provide a detailed description of each core component.

### 2.1. Command Line Interface

We designed and implemented an automated and streamlined interface to facilitate the straightforward usage of our radiomics trend analysis pipeline. This interface accepts pre- and post-condition NIfTI volumes of CT scans as inputs. For the pre- and post-condition states, users can provide either two volume files or two directories containing multiple volumes from different patients, each paired accordingly. Our pipeline generates two main types of output. Firstly, it provides interim results, which include segmentations and extracted radiomic features. Secondly, it offers evaluation results comprising pairwise computed differences for each patient and an overall dynamics analysis for each feature across all patients. 

### 2.2. Structure Segmentation

In radiomics, image segmentation plays a critical role in extracting meaningful features from medical imaging data. Within RadTA, we utilized two segmentation modules for reliable medical image segmentation: TotalSegmentator, developed by Wasserthal et al. [14], and Body Composition Analysis (BCA), developed by Koitka et al. [15].

TotalSegmentator (2023) has demonstrated to be one of the most accurate and reliable segmentation models in the literature and represents the current state of the art in automated body structure segmentation [14]. The segmentation model is based on the deep neural network-based nnU-Net framework [16] trained on 1228 CT scans and incorporates various pre- and postprocessing techniques, like registration and prediction refinement, to enable broad adaptability. TotalSegmentator is capable of segmenting a large number of body structures, including the skeleton, organs, cardiovascular system, gastrointestinal tract, and diverse muscles, resulting in 117 distinct segmentation masks. Moreover, TotalSegmentator offers further expansion by supporting the integration of other models, such as for coronary arteries or lung vessels. This is also fully supported by RadTA. Complementing TotalSegmentator, the BCA model represents our second segmentation module, providing an extensive perspective on the tissue composition within CT scans. This model builds upon the predicted segmentation masks by TotalSegmentator and employs Hounsfield Unit (HU) thresholds to differentiate tissue types, focusing primarily on adipose (−190 to +30 HU) and muscular (−29 to +150) tissues. The segmented adipose tissue is further classified into subcutaneous (SAT), abdominal (VAT), muscular (IMAT), mediastinal (PAT), and pericardial (EAT) regions, resulting in seven segmentation masks, alongside bone and muscle masks. Through categorization based on spinal cord levels (C1–C7, T1–L12, and L1–L5) and whole-body regions (abdominal cavity, thoracic cavity, ventral cavity, mediastinum, as well as pericardium), BCA yields a comprehensive perspective on tissue composition, resulting in 203 segmented tissue masks across 29 body regions in total.

### 2.3. Feature Extraction

In the domain of feature extraction, Haubold et al. [17] recently introduced BOA: Body and Organ Analysis. BOA, released in 2023, incorporates TotalSegmentator and BCA segmentation masks in order to enable the computation of diverse radiomic features. These features consist of various measurements such as volume in milliliters and the statistical properties of Hounsfield units (HU), which are described with eight metrics including the total sum, quartiles (Q1, Q2, Q3), mean, median, minimum, and maximum values for each segmented anatomical structure. These computed metrics align with IBSI standards [18]. Altogether, with the help of BOA, our pipeline is able to extract 2560 radiomic features across 320 body structures (117 by TotalSegmentator and 203 by BCA). 

### 2.4. Preprocessing

As refinement is a crucial step in a radiomic analysis pipeline, RadTA focuses on the inclusion of the only pairwise-existing features present in both pre- and post-condition scans per patient. Non-pairwise-existent features are automatically excluded from further analyses for the corresponding patient. Furthermore, to ensure compatibility and allow the utilization of radiomic features from both segmentation modules, a restructuring process of the radiomic feature table is conducted. The restructuring involves parsing the data into a universal tabular data format that can be expressed in common formats such as CSV, enabling manual insights by users interested in reviewing or analyzing the patient individual radiomic feature tables. This process streamlines the data structure and provides accessible radiomic feature tables for each patient. 

### 2.5. Radiomic Deviation Assessment

Within RadTA, the analysis of radiomic deviation between paired volumes involves computing pairwise absolute and relative differences for each patient. The pairwise absolute differences between pre- and post-condition volumes are computed to directly quantify the magnitude of radiomic feature changes. Additionally, the pairwise relative differences, expressed in percentages, are calculated to provide easier insights into proportional deviation between different radiomic patient profiles. Exceptions are made for cases where a feature is absent in either the pre- or post-condition volume, and if either volume yields a measurement of zero, the relative difference is automatically excluded from any further evaluation or set to zero, respectively. These criteria enhance the reliability of the radiomic trend analyses in the presence of patients when using scans to capture heterogeneous parts of the body. 

### 2.6. Evaluation

Deviation trends for each radiomic feature are automatically evaluated using two methods. As the first evaluation method, macro-averaging of the relative differences is utilized to represent the overall radiomic dynamics across all analyzed patients. Consequently, in the second evaluation method, a dependent t-test for paired samples is applied to directly assess the statistical significance of the observed radiomic dynamics based on the extracted feature scores. The results of the evaluation are presented in a final evaluation table, providing a platform for detailed individual data analysis. Additionally, an overview plot is automatically generated by RadTA, incorporating *t*-test significance as well as relative differences for all evaluated radiomic features, and highlights the relevant radiomic dynamics detected between pre- and post-condition states.

### 2.7. Use Case: Radiomic Health Dynamics in Head and Neck Squamous Cell Carcinoma

For the validation of RadTA, we utilized the HNSCC-3DCT-RT produced by Bejarano et al. [19] from The Cancer Imaging Archive. The authors collected 3D high-resolution CT scans of the head and neck regions of 31 head and neck squamous cell carcinoma patients undergoing radiotherapy. Patients received radiation doses ranging from 58 to 70 Gy. These were administered in daily fractions of 2–2.20 Gy over 30–35 fractions. The dataset contains CT scans at pre-treatment (median 13 days before treatment), mid-treatment (around fraction 17), and post-treatment stages (around fraction 30). The fan-beam CT scans were obtained from a Siemens 16-slice CT scanner, following the standard clinical protocol described in [19]. As an experiment, we applied RadTA to compute the radiomic dynamics between CT scans of pre- and post-treatment. Further patient cohort description is provided in Table 1. 

## 3. Results

**Figure 2:**
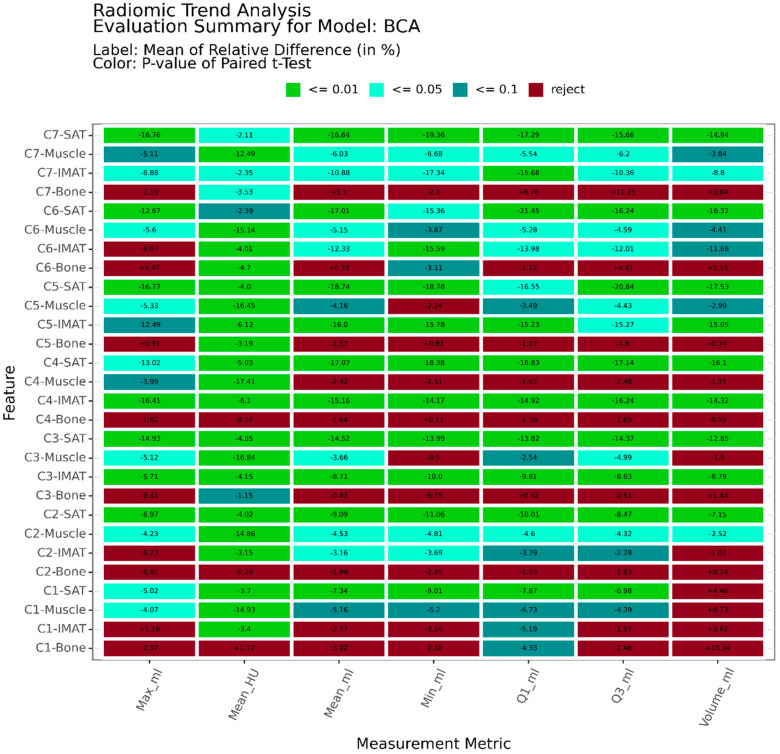
A generated overview visualization for the HNSCC-3DCT-RT dataset produced by RadTa, presenting each evaluated radiomic feature in the cervical spinal cord, its average relative difference, and its deviation significance as color. This is measured by applying a paired *t*-test between pre- and post-treatment radiomic feature scores computed.

We were able to detect several radiomic features, revealing a significant difference between pre- and post-treatment. This is visualized in Figure 2. 

The subcutaneous (SAT) and muscular (IMAT) fat tissue from levels C3 and C7 revealed patient-wide decreases in the total volume of −15.55% and −11.68%, respectively. While the total volumes of bone and muscle did not indicate a clear radiomic trend, it was possible to observe a significant decrease of up to −17.41% in average muscle tissue density (mean HU). With our findings, we were able to confirm that the application of radiation doses has a critical and decreasing impact on fat and muscle tissue in the corresponding head and neck region of a patient, which, as expected, can be attributed to the aggressive nature of radiotherapy. This is consistent with the data analysis [19]. 

### Runtime Performance Analysis of RadTA

**Figure 3:**
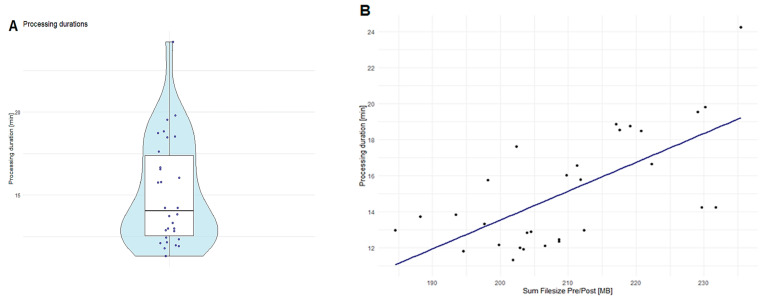
Processing duration analysis results. (**A**): The distribution of processing duration in minutes for the HNSCC-3DCT-RT. (**B**): The relationship between processing time in minutes and file size represented through the file size sum of pre- and post-therapy volumes for the HNSCC-3DCT-RT.

The runtime performance of RadTA was validated using the HNSCC-3DCT-RT dataset, which comprises pairwise head-to-neck CT scans from 31 patients. Through our runtime performance experiment, we identified that the inference of the 320 body structures using the BOA and TotalSegmentator deep neural network models is the most computing-intensive step of our framework. Results from the experiment showed that, on average, RadTA requires 15 min to process a patient, with a standard deviation of 3 min and a runtime ranging from a minimum of 11 min to a maximum of 24 min. Further details of processing duration are illustrated in Figure 3. The hardware resources utilized included an NVIDIA Titan RTX with 24 GB VRAM, an Intel(R) Xeon(R) Gold 5220R CPU @ 2.20GHz, and 32 GB RAM. Notably, it was observed that a GPU VRAM of only 8 GB is sufficient. Moreover, we noted that the overall runtime of RadTA can be drastically improved with increasing numbers of patients through parallelization with multi-processing.

## 4. Discussion

**Figure 4:**
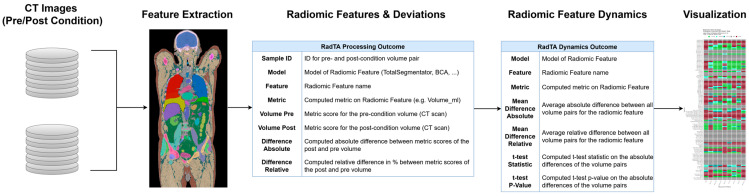
An illustration of the outcomes using the RadTA framework after each implemented step.

We present the RadTA framework for the organ and body feature evaluation based on the timely corresponding CT series of patients. RadTA is designed as an easy-to-integrate CLI application, supporting local deployment in existing applications and workflows. Only a few parameters (e.g., the CT series) and technical requirements are necessary for the application. With such low hurdles, we invite medical experts to add RadTA data-driven features as additional information for decisions in treatment planning. With limited personnel and time resources in CT diagnosis, the findings are focused on pathologies and a specific question; in this case, the regression of the tumor. RadTA’s data-driven approach, on the other hand, offers the possibility of using additional information that is already available from the CT scans, examining it to assess clinical relevance in clinical studies and implementing it in routine diagnostics. Clear feature computation can add a more data-driven approach, thereby supporting medical professionals with useful information, as illustrated in Figure 4. To improve interpretability, our analysis relies on radiomic features and statistical testing rather than black-box deep neural network models. This design choice ensures a closer alignment with clinical practice by providing clear, data-driven insights that clinicians can easily understand and trust. By prioritizing transparency and statistical robustness, our approach addresses key challenges associated with the lack of interpretability in black-box AI systems, a critical factor in fostering trust and promoting the model’s adoption in clinical environments. RadTA offers a visual presentation of the data based on the significance of the calculated trends in order to counteract the AI black box [20] and provide a clear and comprehensible interpretation of the data. Reproducibility was a key focus of this framework’s development. We selected TotalSegmentator and BOA as the core tools due to their popularity, open-source nature, and expected long-term support.

RadTA uses chronological CT series to calculate health dynamics based on the patient’s progression. The HNSCC-3DCT-RT dataset from the Cancer Imaging Archive is used to evaluate the features and performance. The dataset provides CT series taken during pre-treatment, mid-treatment, and post-treatment from oncological patients treated with definitive radiation therapy or concurrent chemoradiation therapy. The measured organ and body feature evaluation shows the drastically visible impact of the therapy on the radiomic features, like the reduction in fat and muscle tissue. The overarching physical degradation could be observed in all patients on the basis of the radiomic features, as stated in the dataset description [19]. Our results are consistent with the current literature, such as the study of Salmanpour et al. [21]. The authors used reproducible radiomics features to predict the survival of head and neck cancer patients. The study shows stable prediction results using various radiological modalities in a traditional oncological use case. However, organ and body feature evaluation is also used in other specialties, as the study by Klontzas et al. [22] demonstrates. The authors use consecutive whole-body CTs to perform the post-mortem interval estimation based on radiomic health dynamics. The prediction results are equal and stable compared to the previously described study and highlight the versatility of organ and body feature evaluation. Abler et al. [23] propose an entire cloud-based system for physician-driven radiomics in order to foster the integration of radiomics tools into the clinical workflow. This system provides a broad stack of functionalities, but consists of various open-source applications and relies on a self-developed PACS. In direct contrast, RadTA has a simpler structure and has no system dependencies that would hinder local integration.

However, it is subject to three limitations. The current implementation only allows a pairwise comparison of CTs. Its extension to multi-series comparison would be desirable—although the benefits are debatable. There are probably only a small number of patients with a high number of CT examinations, as repeated CT scans should be avoided wherever possible due to the associated radiation exposure. With the technical development of computer tomographs and the associated reduction in radiation, shorter examination times and increasing indications, a sharp increase in the number of CT scans is expected in the future. 

The second limitation relates to the comparison of the individual features. The significance of the individual feature trend is determined by a t-test. Since RadTA calculates well over 100 features, it experiences the multiple comparisons problem. As an intuitive countermeasure, RadTA provides two *p*-values (0.01 and 0.05) to allow interpretation. Another approach to counteract this problem is the Bonferroni correction [24], which will be implemented in the future.

The third limitation results from the execution time—this could lead to an application running on a normal PC only as a background process, results are not available ad hoc. As time is one of the most valuable resources in health care systems, high-performance IT systems could solve this issue.

The measured organ and body feature evaluation provided by RadTA shows promising results in the traditional oncological use case, but also enables the support of other clinical scenarios not related to oncology. By automatically registering the images, CT series with a different body window and protocols can be compared. Due to the large number of retrospective radiological images now available [25], new research aspects in other medical specialties can be examined. For example, patients undergoing transcatheter aortic valve implantation receive preprocedural CT scans in planning the procedure. These can be compared with other CT examinations of the patient in question and provide valuable information about the patient’s condition. This could be used, for example, to plan nutritional or physiotherapeutic interventions in the accompanying therapy. 

## 5. Conclusions

The proposed open-source framework RadTA significantly advances radiomics by automating the analysis of patient health dynamics through CT comparisons. Due to the use of advanced segmentation models with comprehensive feature extraction, RadTA provides automatic radiomics trend analysis. Its accessible design targets both clinicians and data scientists and improves clinical usability. The results of the HNSCC-3DCT-RT dataset confirm the effectiveness of the RadTA within an oncological use case but can also be used in other medical fields. RadTA is the first step in integrating machine learning into medical diagnostics and promises a transformative impact and broader clinical applications within patient care. Further research regarding different imaging modalities could further advance the field and could open the door to other clinical applications, e.g., in cardiac magnetic resonance imaging. 

## Figures and Tables

**Table 1:** A summary of demographic and clinical characteristics of the HNSCC-3DCT-RT dataset including patients with head and neck squamous cell carcinoma treated with 3D conformal radiotherapy.

Characteristic		Median (Q1, Q3); n (%)
Patients		31 (100%)
Age		65 (55, 71)
Sex		
	F	8 (26%)
	M	23 (74%)
Cancer staging		
	I	2 (6.5%)
	II	4 (13%)
	III	9 (29%)
	IVA	16 (52%)
PTV volume (cm^3^)		133 (40, 261)
Tumor location		
	Larynx	5 (16%)
	Nose	3 (9.7%)
	Other	8 (26%)
	Tongue	11 (35%)
	Tonsil	4 (13%)
Performance status		
	ECOG 0	12 (39%)
	ECOG 1–2	4 (13%)
	None	15 (48%)
Chemotherapy medication		
	Cetuximab only	2 (6.5%)
	Cetuximab + Cis/Carboplatin	3 (9.7%)
	Cis/Carboplatin only	14 (45%)
	None	12 (39%)

## Data Availability

The code is available under https://github.com/frankkramer-lab/RadTA (accessed on 22 October.2024).
